# A Comparison between Tube Surgery, ND:YAG Laser and Diode Laser Cyclophotocoagulation in the Management of Refractory Glaucoma

**DOI:** 10.1155/2013/371951

**Published:** 2013-10-07

**Authors:** Philip A. Bloom, Colin I. Clement, Anthony King, Baha Noureddin, Kamal Sharma, Roger A. Hitchings, Peng T. Khaw

**Affiliations:** ^1^Western Eye Hospital, 153-173 Marylebone Road, London NW1 5QH, UK; ^2^Sydney Eye Hospital, 8 Macquarie Street, Sydney, NSW 2000, Australia; ^3^Queens Medical Centre, Derby Road, Nottingham NG7 2UH, UK; ^4^Department of Ophthalmology, The American University of Beirut Medical Center, Beirut 113-6044, Lebanon; ^5^Moorfields Eye Hospital, 162 City Road, London EC1V 2PD, UK

## Abstract

*Purpose*. To compare the results of intraocular pressure (IOP) reduction by 3 treatment modalities, (a) glaucoma tube implants, (b) noncontact YAG laser cyclophotocoagulation (cycloYAG), and (c) contact transscleral diode laser cyclophotocoagulation (cyclodiode), in cases of advanced glaucoma refractory to alternative treatments. *Methods*. A consecutive group of 45 eyes that received cycloYAG were matched against two control groups of patients who had received tube surgery or cyclodiode, each control group having been derived from a database of patients. *Results*. Mean pretreatment IOP improved from 41.3, 38.6, and 32.0 mmHg for the tube, cycloYAG, and cyclodiode groups, respectively, to 16.4, 22.1, and 19.3 mmHg, respectively. Treatment success was achieved in 78%, 69%, and 71% of the tube, cycloYAG, and cyclodiode groups, respectively. Visual acuity deteriorated 2 or more Snellen lines in 16%, 7%, and 9% of the patients in the tube, cycloYAG, and cyclodiode groups, respectively. Complications included retinal detachment, hypotony, and phthisis. *Conclusions*. All 3 methods provided acceptable IOP lowering in the short and medium term. Control of IOP was best in patients receiving tube surgery. Cyclodiode and cycloYAG treatments were similarly effective in lowering IOP. Tube surgery was associated with a greater incidence of sight threatening complications.

## 1. Introduction

Treatment options for refractory glaucoma include methods of increasing aqueous outflow, either by antimetabolite augmented trabeculectomy or by the use of aqueous drainage shunts that allow release of fluid from the anterior chamber into the subconjunctival space. Several different types of implants have been used, with varying degrees of success depending on type of implant and diagnosis [1–7].

An alternative approach to lower intraocular pressure (IOP) is to reduce aqueous production from the ciliary body. This was originally carried out using cryotherapy [8], but more recently contact and noncontact transscleral cyclophotocoagulation has been popularised, initially with ND:YAG laser (cycloYAG) and more recently with diode laser (cyclodiode) [9–17]. The use of the diode laser has the theoretical advantage of good penetration and absorption by the tissues of the ciliary body [18–20]. 

In this study the safety and efficacy of cyclodiode were compared to those of both cycloYAG and tube surgery in matched patient groups. The treatments were compared for (1) effect on visual acuity, (2) IOP control, (3) effect of age, (4) subsequent management of IOP control (medical, surgical, and laser), (5) complications, and (6) costs of treatment.

## 2. Methods

Forty-five eyes from 45 patients that underwent tube surgery for refractory glaucoma were matched against 45 eyes from 45 patients receiving cycloYAG and 45 eyes from 45 patients receiving cyclodiode for the same indication. All patients were treated at Moorfields Eye Hospital. “Refractory glaucoma” was defined as glaucoma, which remained uncontrolled despite filtration surgery and maximum tolerated medical treatment. Snellen visual acuity (VA) and IOP were recorded preoperatively and at each postoperative visit. All patients were subject to at least one year of followup.

### 2.1. Tube Group

All tube surgeries were performed between 1990 and 1993 under general anaesthetic using standard techniques. Twenty-seven 2-piece Schocket tubes and 15 one-piece Joseph and 3 double plated Molteno plates were used. All plates were inserted in the superior fornix and secured in place with 9.0 prolene. Tubes were inserted into the anterior chamber between Schwalbe's line and the iris root via a short scleral tunnel fashioned with a 25 G needle. The tube segment between the scleral tunnel and plate was patched with donor sclera.

### 2.2. CycloYAG Group

The cycloYAG treatment was carried out between 1990 and 1993 using a noncontact technique with the free running, thermal mode of the Lasag Microrupter II (Thun, Switzerland). Thirty of the 45 eyes underwent the procedure with local anaesthetic (cocaine 4% drops applied to a cotton-wool pledget placed in the lower fornix). The remainder had retrobulbar anaesthesia. The lower 180° of the ciliary body was treated at the first setting with an average number of 24 applications. The helium-neon beam was aimed at the conjunctiva 1–3 mm from the corneoscleral limbus with maximal offset. The energy used ranged from 3.4 to 5.0 (mean 4.2) Joules.

After cycloYAG patients continued taking all antiglaucoma medications except acetazolamide, which was discontinued. Topical steroids were applied 2 hourly for 2-3 days, and oral analgesia was given as required. Patients were reviewed at 3 days, 2 weeks, and 4 weeks after the procedure and then at 2-month intervals unless otherwise indicated. If the IOP was greater than 25 mmHg with application of topical antihypertensive therapy at 4 weeks the treatment was repeated on the superior 180° with the same settings. A third treatment consisting of 36 burns (energy of 6–8 Joules) applied over 360° was performed if the second treatment failed to control IOP.

### 2.3. Cyclodiode Group

Cyclodiode treatment was carried out between 1993 and 1996 using a continuous wave semiconductor diode emitting at 810 nm, using a transscleral contact “G-Probe” (IRIS Medical Instruments, Inc., Mountain View, CA). Thirty of the 45 eyes underwent the procedure under peribulbar anaesthesia. The remainder had a general anaesthetic. A lid speculum was inserted and the ciliary body was identified using a hand held transilluminator. The heel of the contact probe was placed at the anterior aspect of the ciliary body resulting in application about 1 mm behind this point. The normal treatment consisted of between 20 and 40 applications of 1.5 Watt energy applied over 1.5 seconds from 180° to 360°, and each burn therefore resulted in 2.25 Joules delivered. Following cyclodiode, topical steroids were applied 2–4 hourly for 2 weeks. If the IOP was reduced to below target level, antihypertensive therapy was gradually withdrawn. If the IOP was greater than 25 mmHg at 4 weeks the treatment was repeated up to a maximum of 6 treatments in total.

### 2.4. Matching

Cases were matched based on ranked criteria. This process has previously been described as a retrospective case control study [21], although the alternative term retrospective, nonrandomised study with matched control groups has also been suggested [22].

Cases were ranked first on the basis of diagnostic group and then subsequently for the closest possible match for 5 other successive variables. These were in order of rank: (1) total number of previous operation on study eye, (2) number of previous glaucoma operations on study eye, (3) lens status, (4) vitrectomy status, and finally (5) age in decades (Table 1). Where an acceptable match could not be established for a particular matching tier, cases were then matched for similar characteristics within other diagnostic category groupings. The number of perfect matches therefore decreased during ranking (Table 2). 

The number of patients in each diagnostic category was similar between groups, but exact matches for diagnostic category for matched patients in all three groups were achieved in only 34 cases across the groups. For the remaining 11 patients in each group it was possible to match the diagnosis exactly in 2 of the groups but not the third group. For these remaining 11 cases a diagnostic match was achieved between the cycloYAG-cyclodiode groups but not with the tube group (Table 2). 

Cases were then matched for number of previous operations on the study eye, exact matches being achieved for 14 cases across the groups. Of the remainder, 3 were matched between tube-YAG groups, 4 between tube-diode groups, and 11 between the YAG-diode groups. No exact matching existed for number of previous operations for 13 of the cases across all three groups, but of these patients 10 were matched to within 3 operations and all to within 5 operations.

Exact matches were achieved for number of glaucoma operations on the study eye in 19 cases across the groups. Of the remainder, 5 were matched in the tube-YAG groups, 3 in the tube-diode groups, and 10 in the YAG-diode group. No matching existed for number of glaucoma operations in 8 of the cases. For these cases patients were matched to within 3 operations for 6 patients and within 4 operations for all patients.

Lens status (aphakic/phakic) was matched in 36 cases across the groups. Vitrectomy status was matched in 31 cases across the groups.

Age match was achieved between groups of patients older or younger than 40 years, in 38 cases. When matching by decade (i.e., 21–30 yrs, 31–40 yrs, etc.), age matches within one decade were achieved across 17 cases and matches within 2 decades were achieved in a further 16 cases between the groups (Table 2).

The male to female distribution was 27 : 18 in the tube group, 19 : 26 in the cycloYAG group and 21 : 24 in the cyclodiode group, (*P* = 0.2, *χ* ^2^). To check the similarities of the groups further, they were compared for duration of glaucoma and preoperative IOP levels (Table 3). 

### 2.5. Data Analysis

Analysis of the data determined changes ingroup mean IOPs and median Snellen best corrected visual acuities (VAs) for each of the treatment groups. Patients in each of the 3 treatment groups were then subdivided into those above and below 40 years of age, in order to examine age-related differences within and between treatment groups. Finally, patients in each of the 3 treatment groups were subdivided into those matched by glaucoma diagnosis (postsurgical, POAG, and miscellaneous “others”), to examine diagnosis-related differences within and between treatment groups.

Complications of treatment, the need for further surgical interventions, and the length of hospital stay were compared between treatment groups. Treatment success was defined as IOP less than or equal to 21 mmHg with or without medications at last followup. Partial treatment success was defined as IOP less than or equal to 25 mmHg with or without medication at last followup. Kaplan-Meier analysis was performed to assess IOP “survival” below 22 mmHg, that is, the cumulative probability that IOP would remain below this IOP level following a course of treatment.

For the purposes of the Kaplan-Meier plots, failure of treatment was defined to have occurred if IOP was greater than 21 mmHg on 2 successive visits, having first been at or below this level on 2 successive visits after treatment. The date that treatment failure was said to have occurred was the point in time at which the IOP first rose above 21 mmHg. If IOP never fell below the defined limit or if it fell below this limit only for 1 visit, failure was said to be immediate and the date of failure was the date of the first postlaser measurement of the raised IOP. If IOP was above the set limit only at the last visit, having previously been below this level, this was also defined as a treatment failure for the Kaplan-Meier analysis. This definition of failure is more stringent than simply IOP at last followup, so success rate would be less than on the latter criteria alone. Furthermore the definition is more stringent than that used by many previous authors.

Computerised statistical analysis was performed (SPSS for windows, Version 6.0). Parametric and nonparametric techniques were used as indicated by the data. One-way ANOVA was used to detect differences across the groups; unpaired *t*-test was used for intergroup comparisons. Differences in IOP survival with time, between the groups, were calculated using the logrank test.

Record was made of complications of treatment, the need for further surgical interventions, and the length of hospital stay. The length of hospital stay for procedures performed as “day-cases,” that is, admitted for the procedure and discharged the same day, was recorded as 1 day.

## 3. Results

All patients were followed up at Moorfields Eye Hospital. The mean follow-up time following treatment was 38.2 months (SD = 16.4, range = 12–80), 16.6 months (SD = 6.6, range = 12–40), and 21.5 months (SD = 8.7, range = 4.5–40.8), respectively, for the tube, cycloYAG, and cyclodiode groups.

### 3.1. Intraocular Pressure

**Figure 1:**
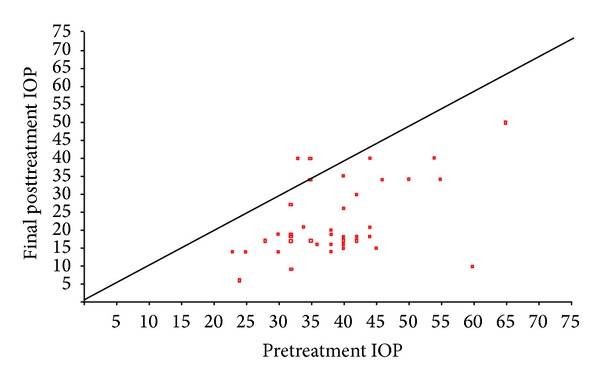
Change in IOP (mmHg) following cycloYAG treatment.

**Figure 2:**
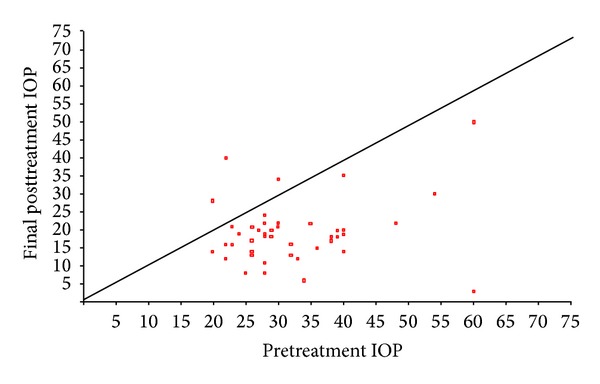
Change in IOP (mmHg) following cyclodiode treatment.

**Figure 3:**
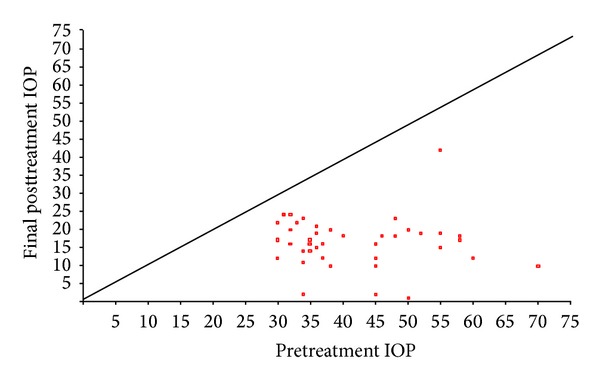
Change in IOP following tube surgery.

**Figure 4:**
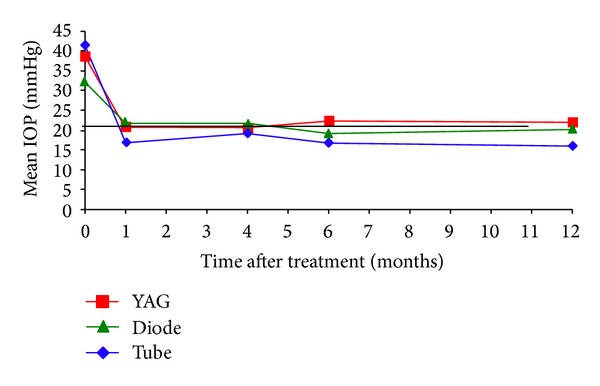
Mean IOP control in the first year following treatment for all groups.

Figures 1, 2, and 3 show the “raw data” for pretreatment IOP and IOP at last followup, for each treatment group. The mean pretreatment IOPs for the tube, cycloYAG, and cyclodiode groups, respectively, were 41.3 mmHg, 38.6 mmHg, and 32.0 mmHg (Figure 4 and Table 3, *P* < 0.00005 between groups). Following treatment there was an immediate IOP reduction in all groups, which reached a plateau about 1 month after treatment. After this, the IOP remained steady in the tube group whereas in the cycloYAG group there was a gradual rise to month 6 after which the level remained steady. In the cyclodiode group there was a further slight drop in IOP until month 6 after which the IOP remained steady (Figure 4).

At the end of followup, the means IOPs were 16.4 mmHg, 22.1 mmHg, and 19.3 mmHg in the tube, cycloYAG, and cyclodiode groups, respectively (Table 3, *P* < 0.007 between groups). This represents a mean IOP reduction of 25 mmHg, 16.6 mmHg, and 12.7 mmHg, respectively (*P* < 0.00005 across groups, *P* < 0.0005 between tube-cycloYAG groups, *P* < 0.0005 between tube-cyclodiode groups, and *P* = 0.09 between cycloYAG-cyclodiode groups), a significant drop in all groups. To overcome the bias introduced by differences in pretreatment IOPs between the groups, the percentage IOP change following treatment was considered by calculating the reduction in IOP at last followup as a percentage of the preoperative IOP. For the tube, cycloYAG, and cyclodiode groups, the IOP was reduced by 58.4%, 42.8%, and 36%, respectively (*P* < 0.0001 across groups, *P* < 0.0005 between tube-cycloYAG groups, *P* < 0.0005 between tube-cyclodiode groups, and *P* = 0.09 between cycloYAG and cyclodiode groups) (Table 3).

The effect of each treatment modality on IOP was also considered by subdividing patients by age (Table 4) and glaucoma diagnosis (Table 5). Reductions in IOP with all 3 treatments were seen in both younger and older patient groups with a greater mean IOP reduction in all treatment groups in the over 40 age group. These differences only reached statistical significance in the cycloYAG group (*P* < 0.03 for the absolute IOP drop, *P* < 0.02 for the % age drop) (Table 4). 

For the purposes examining the effect of each treatment modality on IOP, by glaucoma diagnosis, the diagnostic categories were considered as postsurgical, POAG, and miscellaneous “others” (Table 5). The miscellaneous group represents a mixed group of matched and partially matched patients, so it is difficult to interpret the differences between the groups.

For postsurgical and POAG patients, only cases that were exactly matched across the 3 treatment groups were included in this analysis. There were 12 exact matches across the groups for post-surgical patients. In these 12 patients the biggest mean reduction in IOP was in the tube patients, although none of the differences reached statistical significance. There were 8 exact matches across the groups for POAG patients. In these 8 patients, the greatest mean reduction in IOP was in the tube group and the smallest was in the diode group, with the YAG patients being intermediate (*P* < 0.03 for the absolute IOP drop, Table 5).

### 3.2. Visual Acuity

**Figure 5:**
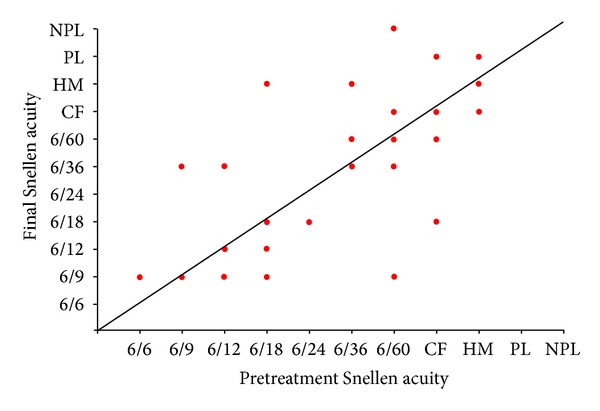
Change in Snellen visual acuity following treatment for Tube group.

**Figure 6:**
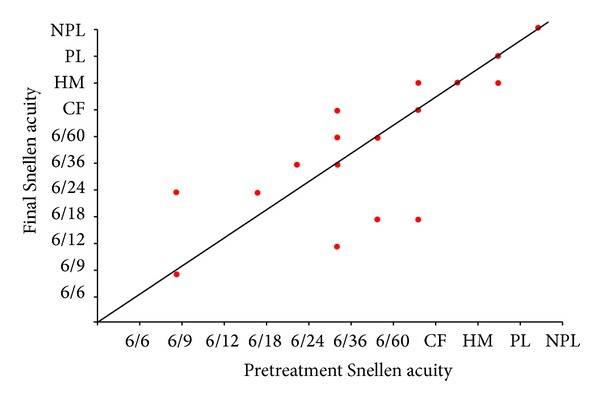
Change in Snellen visual acuity following treatment for cycloYAG group.

**Figure 7:**
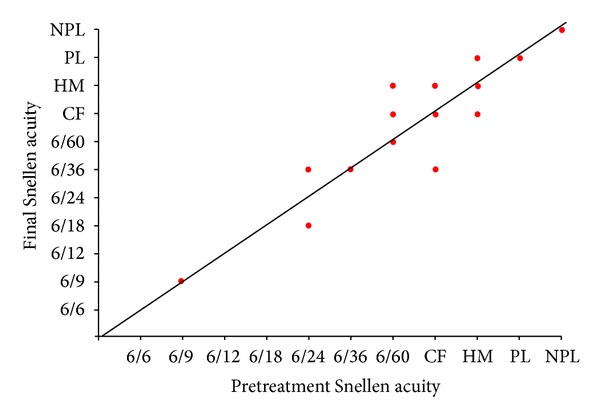
Change in Snellen visual acuity following treatment for cyclodiode group.

Figures 5, 6, and 7 show the “raw data” for pretreatment VA and VA at last followup, for each treatment group. The pretreatment VA varied between 6/6 and HM in the tube group and between 6/9 and NPL in both the cycloYAG and cyclodiode group.

Median pre- and posttreatment VAs for each group are shown in Table 6. There was a difference in the group VAs for both the pre- and posttreatment measurements, with the best VA being in the tube group and the worst being in the cyclodiode group both before and after treatment. No eyes in any group progressed to no light perception which were not already with no light perception before treatment.

Fourteen patients in the tube group and 6 and 8 patients in the cycloYAG and cyclodiode groups, respectively, lost 1 or more lines of Snellen VA. The degree of loss was more severe amongst tube patients with 6 patients losing more than 3 lines compared with only 1 patient in the cycloYAG group and no patients in the cyclodiode group (Table 7).

In an attempt to interpret VA results at low levels of vision, where a Snellen VA could not be recorded, a change of one low vision category (i.e., counts fingers, hand motion, perception of light, or no perception of light) was considered equivalent to 2 consecutive Snellen lines. Using a change of 2 lines of Snellen acuity as the standard for change, after a minimal followup of 12 months, in the tube group 33 (73%) patients remained stable, 5 (11%) improved, and 7 (16%) worsened. The results were 38 (84%), 4 (9%), and 3 (7%), respectively, in the cycloYAG group and 39 (87%), 2 (4%), and 4 (9%), respectively, in the cyclodiode group. There were no statistical differences between the groups for change in VA, ((*P* = 0.7), Kruskal-Wallis one-way ANOVA) (Table 6).

### 3.3. Other Results

Complications associated with the 3 different treatments are enumerated in Table 8. Sight threatening complications following treatment were more common in the tube group. Serious complications included corneal touch, retinal detachment, choroidal detachment, expulsive haemorrhage, vitreous incarceration, and endophthalmitis.

Complication common to all treatment modalities included vitreous haemorrhage and hypotony (defined as 0 mmHg < IOP < 5 mmHg). Phthisis developed in 1 patient only, in the tube group. Chemosis, anterior uveitis, and pain were the commonest complications in the cycloYAG and cyclodiode groups.

Further surgical intervention was required in all groups, with the tube group requiring most intervention (33%). These interventions are summarised in Table 9.

Mean length of hospital stay was longer for the tube group than either for the other 2 groups, (*P* < 0.0005, Table 10). The majority of cycloYAG and cyclodiode patients were either treated as day-cases or stayed in hospital overnight after the treatment.

All groups had patients requiring retreatment; for the tube group the mean number of treatments was 1.1 (SD = 0.38, range 1–3), for the cycloYAG group the mean number of treatments was 1.62 (SD = 0.75, range 1–4), and for the cyclodiode group the mean number of treatments was 1.9 (SD = 1.2, range 1–7).

The figures for treatment success, as defined in “Section 2”, were 78% in the tube group (7 IOP > 21 mmHg and 3 IOP < 5 mmHg), 69% in the cycloYAG group (14 IOP > 21 mmHg and 0 IOP < 5 mmHg), and 71% in the cyclodiode groups (12 IOP > 21 mmHg and 1 IOP < 5 mmHg) at the end of followup (*P* = 0.45, Kruskal-Wallis one-way ANOVA). The figures for partial treatment success were 91% (1 IOP > 25 mmHg and 3 IOP < 5 mmHg), 69% (14 IOP > 25 mmHg and 0 IOP < 5 mmHg), and 84% (6 IOP > 25 mmHg and 1 IOP < 5 mmHg), respectively, for the three groups at the end of followup (*P* = 0.02, Kruskal-Wallis one-way ANOVA).

**Figure 8:**
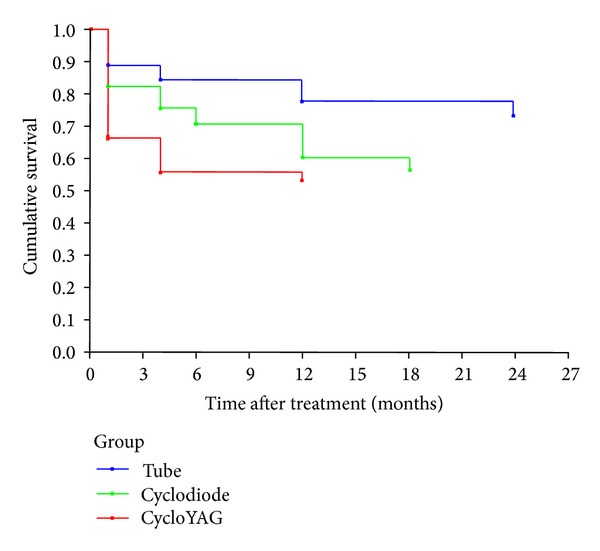
Kaplan-Meier curves showing IOP control following treatment for all groups.

Kaplan-Meyer survival analysis shows IOP reduction to be maintained better with tube than with either cycloYAG or cyclodiode (Figure 8). Only the difference in IOP survival between the tube group and the cycloYAG group was statistically significant (*P* < 0.02).

## 4. Discussion

Refractory glaucoma presents a difficult management problem. A variety of methods exist for the treatment of patients in whom initial medical, laser, and surgical treatments have been unsuccessful in lowering IOP and thereby halting the progression of glaucomatous damage. Drainage procedures, such as antimetabolite augmented trabeculectomy, are a potential solution but may be associated with a number of complications, including hypotony, leaking blebs, and endophthalmitis [23–26]. Aqueous tube shunt procedures have been shown to be an effective method of IOP control [1–7], but their use may also be fraught with difficulties, as illustrated by the results above.

Cyclodestruction is an older method of IOP control. Until the 1990s this was not widely used because of the high incidence of phthisis and sympathetic ophthalmitis with relatively blunt treatment modalities such as cyclocryotherapy [27, 28]. Since then, however, more precise and less destructive modes of cycloablative treatment have become available. These methods include external methods such as contact and noncontact YAG or diode laser cyclophotocoagulation and more recently still treatment via direct endoscopic ciliary body photocoagulation. All of these methods have been reported to achieve reasonable IOP control [18, 29–33].

The decision on whether to treat a refractory glaucoma with tube surgery or cyclophotocoagulation is not straightforward. Factors that influence the choice include reason for treatment, visual potential, prior treatments, glaucoma type, surgeon preference, and patient preference. A further important consideration is likelihood of success. In this regard, little is known on the comparative benefits of tube surgery versus cyclophotocoagulation for refractory glaucoma. We have attempted to address this issue by comparing the outcomes of tube surgery with cycloYAG or cyclodiode in this setting. This study shows that larger IOP reduction can be achieved with tube surgery compared to either cyclophotocoagulation technique. However, significantly more patients receiving tube surgery lost vision after treatment, and the rate of postoperative complications was higher in this group overall. Importantly, the rate of hypotony and/or phthisis bulbi was low in all treatment groups.

We are aware of at least 3 studies that have examined the IOP response and complications of transscleral cyclophotocoagulation or tube surgery in refractory glaucoma. Most recently, Yildirim et al. [34] prospectively assessed cyclodiode or Ahmed tube surgery for neovascular glaucoma. Overall, no significant difference was found between groups: 24-month success probability was 61.18% and 59.26%, respectively. Similar results have been reported by Noureddin et al. [22] when comparing noncontact cycloYAG to tube surgery for refractory glaucoma. In contrast, a small retrospective study [35] with only 6-month followup suggests that outcomes in neovascular glaucoma are better with pars plana Baerveldt tube surgery compared to cycloYAG.

Differences between the present study and those described above make direct comparison challenging. The type of tube surgery performed differs both in type (Baerveldt versus Ahmed versus other) and location (anterior segment versus pars plana) used. Some studies looked exclusively at neovascular glaucoma [34, 35], whilst others studied a heterogenous population [22]. It is also true that, in general, cyclophotocoagulation techniques are nonstandardised: this may influence efficacy. For these reasons, it is perhaps not surprising that different outcomes have been reported. The findings of Chalam et al. [35] are similar to those of this study in that tube surgery was associated with larger mean IOP reduction whereas the 2 other studies found equivalent IOP reduction between tube surgery and cyclophotocoagulation techniques. Interestingly, despite the many differences, no study has yet found larger mean IOP reductions with cycloYAG or cyclodiode.

Because of the significant differences outlined above, clinicians are left to examine results of completely unmatched and noncomparable studies to assess the relative pros and cons of each treatment. To address this issue, we have employed a novel study design, attempting to control for some of the variables that may confound comparison between individual reports of the efficacy of different treatments but without the need for a prospective trial. This method provides valuable insights into treatment options for patients with refractory glaucoma, as long as the limitations of the method are constantly borne in mind.

Of the 3 treatment arms in this study, all modes of therapy result in a significant drop in baseline IOP. The extent of this drop was found to differ significantly between the groups. The comparison was complicated, however, by inequality in the baseline IOPs between groups. Some of the differences may be explained by increased experience and confidence in cyclodestruction, leading to lower IOP threshold for listing patients for this treatment modality; this is a weakness of this study design.

In an attempt to eliminate some of the bias that may be introduced by the different baseline IOP levels, the percentage IOP drop was considered. Examination of the percentage IOP reduction between groups revealed no statistical difference between the two laser groups but a significantly greater IOP reduction in the tube than in both laser groups. This finding that tube implantation offers the greatest IOP reduction is further supported by the final IOP levels, overall success rates, and the results of the Kaplan-Meier analysis of IOP survival.

Interpretation of these study results is further complicated by the surgical techniques used in the tube group. Both the Schocket procedure and Joseph tube have been largely superseded by more widely adopted tube techniques. It could be argued that comparison of this surgical group to cyclodiode or cycloYAG is not relevant in the modern era. This is because modern tube surgery techniques have been adopted in preference to the Schocket procedure or Joseph tube because of perceived improved efficacy, better complication profile, and ease of use. However, a study comparing the outcome of the Schocket procedure with the double-plate Molteno implant for refractory glaucoma found that this is not the case [36]. After a mean followup of 26 months, intraocular pressure had reduced from 35.2 mmHg to 15.1 mmHg in the Schocket group and from 34.6 mmHg to 14.4 mmHg in the Molteno group. At final followup, the number of medications was lower in the Schocket group (0.43 versus 0.95), but stable or improved visual acuity was observed more often in the Molteno group (48% versus 68%). We are not aware of any studies comparing Schocket tube surgery to Baerveldt, or Ahmed implants, nor have there been any direct comparisons of Joseph tubes with Molteno, Baerveldt, or Ahmed tube surgery. Interstudy comparison suggests that pressure reduction is similar between the older and newer techniques but that newer tubes better preserve visual function and have a lower rate of complications.

That the tube group largely comprised eyes receiving Schocket or Joseph tubes does not detract from the fundamental observations of this study. The aim was to compare the outcome of inflow versus outflow treatment in refractory glaucoma. We found that success was statistically equivalent between groups but that complications occurred more frequently after the outflow procedure (tube surgery). 

It has previously been noted that patients younger than 40 years of age have a worse response to laser cyclophotocoagulation treatment than their older counterparts [14]. Within both laser groups our results suggest confirmation of this finding; there was a statistically significant difference between the older and younger groups in final IOP, with the older groups having the better response. Only in the cycloYAG group was there demonstrated a statistically significant difference between older and younger patients in terms of the percentage IOP drop after treatment, with the older patients responding better.

In terms of glaucoma diagnosis in this study, the 2 largest exactly matched subgroups were the postsurgical and POAG groups. Comparison of the different modes of treatment in these subgroups showed no difference in success for the postsurgical group, while in the POAG group the magnitude of the IOP drop was greater in the tube group than in either laser group (*P* < 0.03). However, these results are confounded by small numbers of exactly matched patients in each subgroup.

The visual acuity of patients with refractory glaucoma is often poor, and the analysis of changes in vision after any treatment is problematic. Visual loss after treatment may be as a direct complication of the treatment, or as an indirect complication if there is glaucoma progression due to a treatment failing adequately to control IOP. Furthermore, it is difficult to statistically analyse differences in Snellen visual acuity following treatment, especially at low levels of vision.

In the present study, a number of patients lost some vision after treatment in all 3-treatment groups; the proportion of patients affected and the severity of visual loss appeared worst in the tube group. One possible explanation for this is the better pretreatment VA in the tube group, this group therefore having more potential visual acuity to lose. However no patients went to NPL from a better level of VA. Furthermore, the fact that posttreatment IOP was lowest in the tube group means that any observed changes in vision was less likely to be due to glaucoma progression and more likely to be as a direct complication of the treatment. It should be noted that a recent report of transscleral cyclodiode in eyes with difficulty to control glaucoma yet good visual acuity found that while 30.6% lost more than 2 Snellen lines of acuity post treatment, the remainder maintained good acuity over the longterm [37].

Complications in general, as well as sight-threatening complications in particular, were most common in the tube treated group and least common in the cyclodiode group, which causes concern over the use of tube treatments. Posttreatment surgical intervention was also most common in the tube group and least common in the cyclodiode group.

All patients in the tube group required a general anaesthetic and a relatively prolonged postoperative hospital stay, whereas the majority of patients in both laser groups had treatment carried out under local anaesthesia requiring minimal hospital stay. Since the time of the study, inpatients lengths of stay have decreased, but there is little doubt that tube surgery is a more complicated, time-consuming, and resource/finance intensive treatment than cyclodestruction.

## 5. Conclusion

In conclusion this retrospective study attempts to match 3 modalities of treatment for the management of refractory glaucoma. All 3 methods are successful in producing IOP reduction, but there is little doubt that the greatest lowering of IOP can be achieved with tube treatment. Both laser treatments achieved similar IOP results; the absolute and percentage IOP reductions were slightly greater in the cycloYAG group, but the IOP survival analysis slightly favoured cyclodiode treatment. Patients in the tube group, however, developed the most complications after treatment, and a number of these were potentially sight threatening. In addition, patients in the tube group required the most surgical reintervention and stayed in hospital longer immediately following treatment.

The preservation of visual acuity, relative lack of posttreatment complications, and the easy repeatability of modern methods of cyclophotocoagulation make such procedures useful alternatives to tube implantation in the armamentarium of treatments against refractory glaucoma, although the reduction in IOP may not be as marked. In this study, the complication profile of the cyclodiode laser was better than that of cycloYAG. In addition, the diode laser is more convenient, portable, and more widely available than cycloYAG. In our unit, cyclodiode is therefore the cyclodestructive modality of choice. Future research should focus on comparison between transcleral cyclodiode and modern tube surgery techniques with an emphasis both on efficacy and costeffectiveness.

## Figures and Tables

**Table 1:** Matching criteria for the three groups of patients, showing the number of patients in each of the groups with each of the ranking variable values.

	CycloYAG	Cyclodiode	Tube
Diagnosis			
POAG	11	11	11
Postsurgical	14	14	14
Posttrauma	4	4	5
Inflammatory	4	4	3
CACG	3	3	3
Aniridia	2	2	3
Buphthalmos	2	2	2
Juvenile	2	2	1
Neovascular	1	1	1
Other	2	2	2
Total	**45**	**45**	**45**
Mean number of previous operations			
None	3.1	2.7	3.2
1 operation	7	7	2
2 operations	5	5	9
3 operations	8	12	13
>3 operations	7	9	5
Mean number of previous glaucoma ops.	1.5	1.3	1.6
None	20	19	13
1 operation	8	10	15
2 operations	5	9	7
3 operations	5	3	5
>3 operations	7	4	5
Aphakia	34	30	35
Vitrectomised	13	10	11
Mean age in yrs (SD)	48.6 (20.7)	49.8 (19.8)	44.2 (17.7)

POAG: primary open angle glaucoma, CACG: chronic angle closure glaucoma, and diagnosis was not always matched with similar diagnoses in other groups; see Table 2.

**Table 2:** Accuracy of matching across patient groups and for group pairs, *n* = 45 for each group.

Patient group pairs	All groups	Tube/YAG	Tube/diode	YAG/diode
Matching criteria	Exact matches (%)	Exact matches (%)	Exact matches (%)	Exact matches (%)

Glaucoma diagnosis	34 (75.5)	34 (75.5)	34 (75.5)	45 (100)
Previous operations	14 (31.1)	17 (37.7)	18 (40.0)	25 (55.5)
Glaucoma operations	19 (42.2)	24 (53.3)	22 (48.8)	29 (64.4)
Lens status	36 (80.0)	42 (93.3)	36 (80.0)	39 (86.6)
Vitrectomy status	31 (68.8)	32 (71.1)	35 (77.7)	40 (88.8)
Age	38 (84.4)	40 (88.8)	39 (86.6)	38 (84.4)

**Table 3:** Clinical characteristics of the three groups of patients (standard deviation).

	CycloYAG	Cyclodiode	Tube	*P*
Male : female	19 : 26	21 : 24	27 : 18	0.02^□^
Preoperatively				
Mean duration of glaucoma (months)	97.7 (72.8)	116.0 (102.9)	102.2 (90.1)	NS
Mean preop IOP	38.6 (8.7)	32.0 (9.5)	41.3 (9.7)	<0.00005*
Postoperatively				
Mean duration of followup (months)	16.6 (6.4)	21.5 (8.7)	38.2 (16.4)	<0.0005^□^
Mean final postop IOP	22.1 (9.9)	19.3 (8.5)	16.4 (6.6)	<0.007^§^
Mean IOP drop	16.6 (9.5)	12.7 (11.4)	25.0 (11.9)	<0.00005^□^
Mean % IOP drop	42.8 (21.7)	36.0 (30.1)	58.4 (18.2)	<0.0001^□^

IOP: intraocular pressure (mmHg), NS: no statistically significant difference, ^□^tube group differs statistically significantly from both cycloYAG group and cyclodiode group, *cyclodiode group differs statistically significantly from both tube group and cycloYAG group, ^§^statistically significant difference between tube group and cycloYAG group.

**Table 4:** Effect of treatment on IOP for each patient group, by age. IOP drop is from preop to last followup.

	CycloYAG			Cyclodiode			Tube			ANOVA	
	<40	>40	*P*	<40	>40	*P*	<40	>40	*P*	*P* < 40	*P* > 40
Age	24.2	60.7	—	26.9	62.4	—	26.6	57.0	—	NS	NS
Preop IOP	38.8	38.5	NS	30.9	34.0	NS	38.0	43.8	0.04	NS	0.0001*
12 M IOP	27.2	19.4	0.02	24.3	17.7	0.04	16.5	15.4	NS	0.005^§^	NS
Final IOP	27.2	19.6	0.02	23.3	17.2	0.04	16.8	16.0	NS	0.01^§^	NS
IOP drop	11.6	18.9	0.03	10.8	13.7	NS	21.2	27.8	0.06	0.007^□^	0.0001^□^
% IOP drop	29.4	49.2	0.02	27.5	40.7	NS	54.8	61.0	NS	0.007^□^	0.001^‡^

NS: no statistically significant difference, ^□^tube group differs statistically significantly from both cycloYAG group and cyclodiode group, *cyclodiode group differs statistically significantly from both tube group and cycloYAG group, ^§^statistically significant difference between tube group and cycloYAG group.

**Table 5:** Table showing comparisons for matched categories. Exact matches were achieved for all category ranks in 12 of the postsurgical group and 8 of the POAG group. The miscellaneous group represents patients who are mismatched in one or more categories. IOP drop is from preop to last followup.

	Postsurgical (*n* = 12)				POAG (*n* = 8)				Miscellaneous (*n* = 25)			
	T	Y	D	*P*	T	Y	D	*P*	T	Y	D	*P*
Preop IOP	41.4	37.8	33.8	NS	42.6	40.6	32.6	NS	40.9	38.3	30.9	<0.0004
12 M IOP	19.8	23.8	20.7	NS	17.5	21.8	21.0	NS	13.7	21.3	19.6	0.003
Final IOP	19.8	23.4	19.8	NS	16.8	22.5	20.3	NS	14.6	18.8	21.4	<0.02
IOP drop	21.7	14.4	13.9	NS	25.9	18.1	12.4	0.03	26.3	16.9	12.2	<0.0005
% IOP drop	49.9	37.8	35.2	NS	58.5	44.3	37.1	NS	62.5	44.4	36.0	0.001

NS: no statistically significant difference, T: tube group of patients, Y: cycloYAG group of patients, and D: cyclodiode group of patients.

**Table 6:** Pre- and posttreatment visual acuities for each group. Changes in VA category represent 2 or more lines of Snellen acuity change.

Group	Median pre-Rx VA (range)	Median post-Rx VA (range)	↑VA	↓VA	Stable VA
CycloYAG	CF (6/9-NPL)	CF (6/9-NPL)	4	3	38
Cyclodiode	CF (6/9-NPL)	CF (6/9-NPL)	2	4	39
Tube	6/60 (6/6-HM)	6/60 (6/6-HM)	5	7	33
*P* value	0.004	0.04	0.695		

Statistical significance *P* < 0.05 (Kruskal-Wallis one-way ANOVA).

**Table 7:** Loss of VA following treatment.

Snellen visual acuity	CycloYAG	Cyclodiode	Tube
*Total pts loss* = 1* line *	**6**	**8**	**14**
Total loss = 3 line	**1**	**0**	**6**
Retinal detachment	1		3
Corneal decompensation			3
Total loss = 2 line	**1**	**1**	**1**
Phthisis			1
PK decompensation	1		
Corneal decompensation		1	
Total loss = 1 line	**4**	**7**	**7**
Corneal decompensation	1	2	2
Uncontrolled IOP	1	4	0
PK decompensation	0	1	2
Cataract	0	0	2
Vitreous haemorrhage	0	0	1
Retinal detachment	1	0	0
Uveitis	1	0	0

PK: penetrating keratoplasty, IOP: intraocular pressure.

**Table 8:** Complications of the three treatment modalities.

Complication	CycloYAG (%)	Cyclodiode (%)	Tube (%)
Vitreous incarceration	—	—	10 (22)
Corneal touch	—	—	10 (22)
Conjunctival dehiscence	—	—	8 (18)
Fibrosis over plate	—	—	5 (11)
Choroidal detachment	—	—	4 (9)
Retinal detachment	1 (2)	—	4 (9)
Progression of cataract	—	—	3 (7)
Expulsive haemorrhage	—	—	2 (4)
Vitreous haemorrhage	2 (4)	1 (2)	1 (2)
Endophthalmitis	—	—	1 (2)
Hypotony	—	1 (2)	3 (2)
Phthisis	—	—	1 (2)
Chemosis	31 (69)	3 (7)	—
Anterior uveitis	25 (56)	4 (9)	—
Pain	17 (38)	4 (9)	—
Corneal oedema	8 (18)	2 (4)	—
Vitritis	7 (16)	—	—
Hyphema	7 (16)	1 (2)	—
Hypopyon uveitis	3 (7)	—	—
IOP spike	1 (2)	—	—
Macular oedema	1 (2)	—	—
PK decompensation	—	1 (2)	—

**Table 9:** Number of patients requiring posttreatment intervention, *n* = 45 for each group.

	CycloYAG	Cyclodiode	Tube
Procedure			
Repositioning of tube			5 (11%)
Repair of conjunctival dehiscence			3 (7%)
Insertion of new tube	3 (7%)	2 (4%)	4 (9%)
Release of fibrosis			2 (4%)
Cyclocryotherapy	6 (13%)		2 (4%)
Trabeculectomy/vitrectomy	1 (2%)		
Repeat of original procedure	22 (49%)	24 (53%)	4 (9%)

Total	10 (22%)	2 (4%)	15 (33%)

**Table 10:** Mean inpatient treatment time for the three groups of patients. A “day-case” admission was recorded as staying in hospital for 1 day.

	Mean hospital stay (SD, range)
CycloYAG patients	1.4 days (0.8, 1–10 days)
Cyclodiode patients	1.2 days (0.6, 1–3 days)
Tube patients	4.8 days (1.6, 1–10 days)
*P* value	<0.0005

Statistical significance *P* < 0.05 (Kruskal-Wallis one-way ANOVA).
